# CW-NET for multitype cell detection and classification in bone marrow examination and mitotic figure examination

**DOI:** 10.1093/bioinformatics/btad344

**Published:** 2023-05-30

**Authors:** Ching-Wei Wang, Sheng-Chuan Huang, Muhammad-Adil Khalil, Ding-Zhi Hong, Shwu-Ing Meng, Yu-Ching Lee

**Affiliations:** Graduate Institute of Biomedical Engineering, National Taiwan University of Science and Technology, Taipei City, 106335, Taiwan; Graduate Institute of Applied Science and Technology, National Taiwan University of Science and Technology, Taipei City, 106335, Taiwan; Department of Laboratory Medicine, National Taiwan University Hospital, Taipei, 100225, Taiwan; Department of Hematology and Oncology, Hualien Tzu Chi Hospital, Buddhist Tzu Chi Medical Foundation, Hualien, 970473, Taiwan; Department of Clinical Pathology, Hualien Tzu Chi Hospital, Buddhist Tzu Chi Medical Foundation, Hualien, 970473, Taiwan; Graduate Institute of Applied Science and Technology, National Taiwan University of Science and Technology, Taipei City, 106335, Taiwan; Graduate Institute of Biomedical Engineering, National Taiwan University of Science and Technology, Taipei City, 106335, Taiwan; Department of Laboratory Medicine, National Taiwan University Hospital, Taipei, 100225, Taiwan; Graduate Institute of Applied Science and Technology, National Taiwan University of Science and Technology, Taipei City, 106335, Taiwan

## Abstract

**Motivation:**

Bone marrow (BM) examination is one of the most important indicators in diagnosing hematologic disorders and is typically performed under the microscope via oil-immersion objective lens with a total 100× objective magnification. On the other hand, mitotic detection and identification is critical not only for accurate cancer diagnosis and grading but also for predicting therapy success and survival. Fully automated BM examination and mitotic figure examination from whole-slide images is highly demanded but challenging and poorly explored. First, the complexity and poor reproducibility of microscopic image examination are due to the cell type diversity, delicate intralineage discrepancy within the multitype cell maturation process, cells overlapping, lipid interference and stain variation. Second, manual annotation on whole-slide images is tedious, laborious and subject to intraobserver variability, which causes the supervised information restricted to limited, easily identifiable and scattered cells annotated by humans. Third, when the training data are sparsely labeled, many unlabeled objects of interest are wrongly defined as background, which severely confuses AI learners.

**Results:**

This article presents an efficient and fully automatic CW-Net approach to address the three issues mentioned above and demonstrates its superior performance on both BM examination and mitotic figure examination. The experimental results demonstrate the robustness and generalizability of the proposed CW-Net on a large BM WSI dataset with 16 456 annotated cells of 19 BM cell types and a large-scale WSI dataset for mitotic figure assessment with 262 481 annotated cells of five cell types.

**Availability and implementation:**

An online web-based system of the proposed method has been created for demonstration (see https://youtu.be/MRMR25Mls1A).

## 1 Introduction

Examination of bone marrow (BM) is crucial for the diagnosis and management of many disorders of the blood and BM (Lee *et al.* 2008). BM nucleated differential cell count (NDC) is compulsory to assess the hematopoiesis in different cell lineages and the proportion of aberrant cells. That is, BM NDC is an invaluable assessment that not only produces a correct diagnosis but also provides a significant indicator of prognosis and disease follow-up, particularly for hematological malignancies like acute myeloid leukemia (Greenberg *et al.* 2012), chronic myeloid leukemia (Swerdlow *et al.* 2017) and multiple myeloma (Kumar *et al.* 2016). Compared with general pathological examinations, which usually identify only one or a few types of tumor tissues for each analysis, BM NDC analysis is much more complicated and difficult as there are >16 types of cells to be detected and classified at once. In addition, for pathological diagnosis, pathologists may conduct a microscopic assessment on WSIs using computer-assisted systems (Campanella *et al.* 2019), but for BM examination, BM NDC analysis is generally conducted via oil-immersion objective lens with a total 1000× magnification that makes fully automated analysis more challenging. Apart from the diversity of cell types, challenges include delicate intralineage discrepancy within the BM cell maturation process, cells overlapping, lipid interference and stain variation, causing large intra- and interobserver variability (Chandradevan *et al.* 2020). The enormous size of WSIs makes automated BM NDC analysis on WSIs more difficult. In addition, a previous study shows that existing modern hematology analyzers are poor in recognition and detection of blasts, immature granulocytes and basophils (Meintker *et al.* 2013). In practice, BM NDC analysis requires well-trained examiners to perform cytomorphological assessment intensively from low to high magnification (×10, ×40 up to ×100 objective magnification with oil immersion). According to International Council for Standardization in Hematology guidelines (Lee *et al.* 2008), in order to generate percentages of the number of required cell types for diagnosis and disease, at least 500 cells should be analyzed on each smear and at least two smears are assessed for each patient. An accurate and reliable BM NDC analysis system is highly demanded in order to improve diagnostic precision, speed and reliability and to minimize valuable human resource costs. In this study, we build a large WSI dataset with 16 456 annotated cells of 19 BM cell types to evaluate the robustness and generalizability of the proposed model.

Early screening and diagnostic information will aid in lowering death rates and in better understanding the aggressiveness of cancer stages. The Nottingham Grading System (NGS) is commonly used to grade three major tumor features: tubule development, nuclear pleomorphism and mitotic rate (Balkenhol *et al.* 2019), and according to the NGS, the mitotic rate has the highest predictive value among the three (Balkenhol *et al.* 2019). Hence, mitotic detection and identification are critical not only for accurate cancer diagnosis and grading but also for predicting therapy success and survival (Bray *et al.* 2018). Pathologists often do such mitotic identification tasks visually, which is time-consuming, subjective and poorly reproducible with considerable inter- and intra-rater variability due to the difficulties in recognizing mitotic figures and their varied distribution across WSIs (Bertram *et al.* 2020). Previous studies have found 17.0% to 34.0% inter-rater disagreement in distinguishing individual mitotic figures from other cell features in the canine cutaneous mast cell tumor and human breast cancer (Malon *et al.* 2012, Bertram *et al.* 2020). As a result, developing an automated computer-aided approach for mitotic figure examination is highly demanded. In recent years, there have been a number of international medical image analysis challenges in the field of automatic identification of mitotic figures, such as MIDOG 2021 challenge (Aubreville *et al.* 2023), TUPAC16 challenge (Veta *et al.* 2019), ICPR MITOS-ATYPIA-2014 challenge (Roux 2014) and ICPR MITOS-2012 challenge (Ludovic *et al.* 2013). However, the number of annotated mitotic figures in these datasets is small (fewer than one thousand annotated cells for each dataset). To evaluate robustness and generalizability of the proposed method, we utilized a large-scale WSI dataset (Bertram *et al.* 2019) with 262 481 annotated cells of five cell types for mitotic figure assessment.

**Figure 1.**
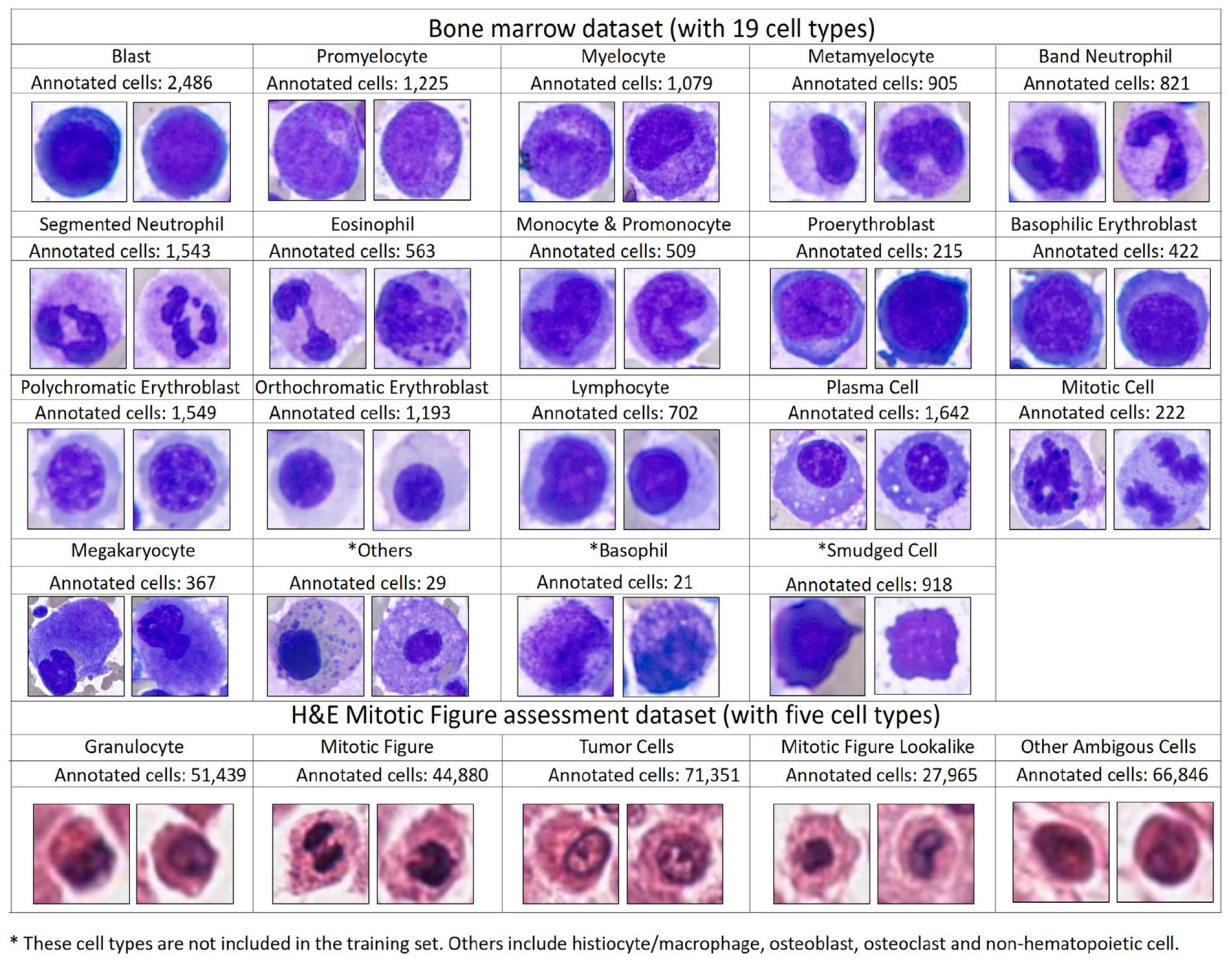
Sample cells with the number of the annotated cells of various cell types in the two datasets used in this study

In this study, we present an effective, fully automatic and fast deep learning approach (CW-Net) for multitype cell detection and classification in BM examination and mitotic figure examination. The proposed weakly supervised learner is demonstrated to be useful for applications with partially annotated data and for boosting up the model performance in both object detection and classification. The rest of this article is organized as follows. Related works on weakly supervised learning, (semi)-automatic BM analysis and (semi)-automatic mitotic figure examination are described in Supplementary Section S1. Figure 1 presents sample cells of various cell types in the two datasets used in this study; see Supplementary Section S2. Section 2 describes the proposed method. The experimental results in comparison with the benchmark methods are given in Section 3. Section 4 concludes the article.

## 2 Materials and methods

### 2.1 Proposed CW-Net

When the training data are partially labeled, causing many unlabeled objects of interest wrongly defined as background or contents of no interest, this severely confuses AI learners during supervised learning and deteriorates the performance of output AI models. The proposed deep learning approach is devised with (i) a Jaccard-based soft sampling weighted loss function to achieve a reasonable balance between hard examples and the rest of the background in instance sampling, (ii) a dual layer filtered negative instance sampling (FNIS) strategy to generate better detectors and classifiers, (iii) a multiclass nonmaximum-suppression (MCNMS) strategy to ensure no contradictory prediction of a cell and (iv) a data augmentation and normalization strategy to minimize generalization errors and prevent overfitting.

**Figure 2.**
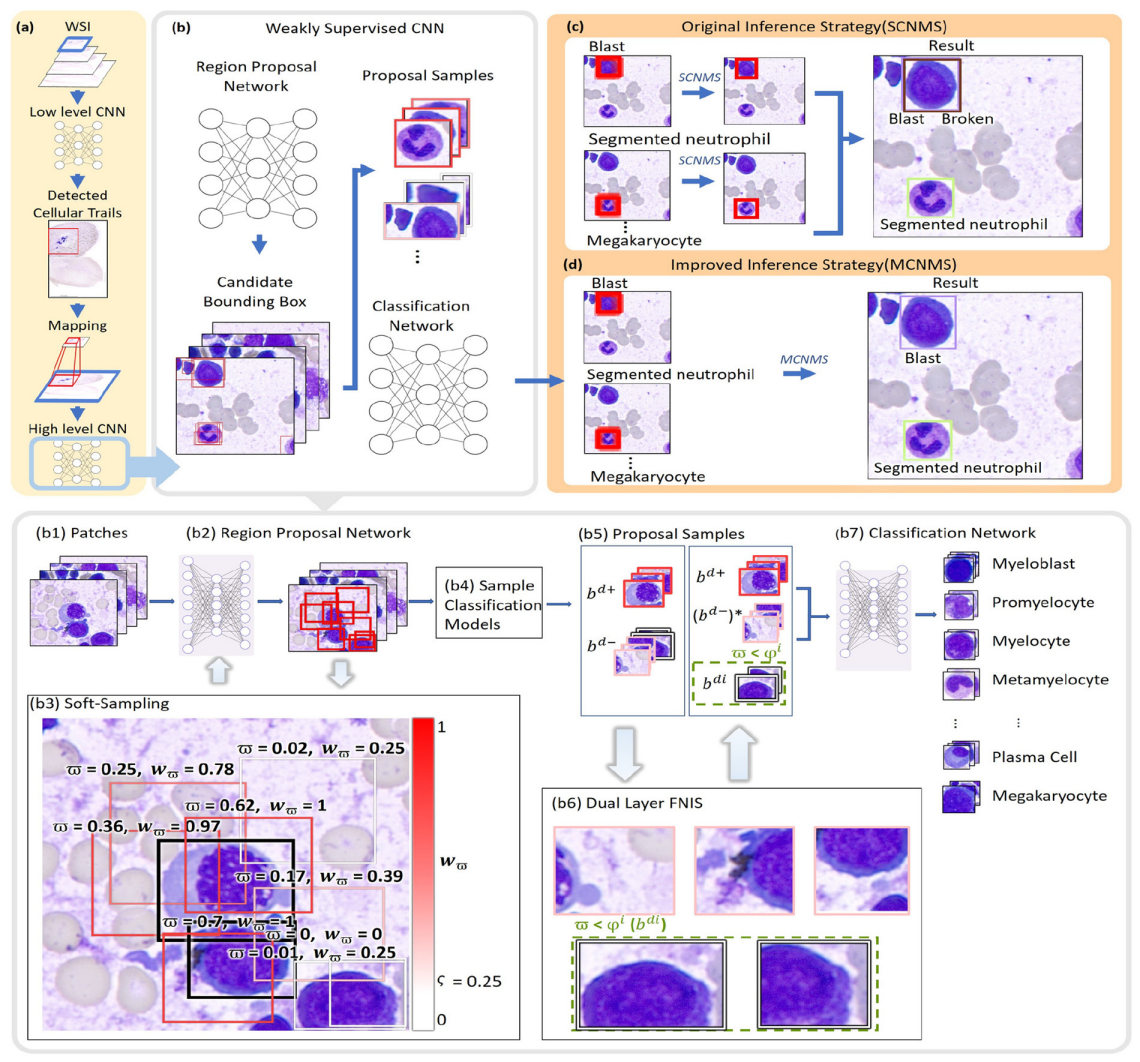
The proposed deep learning framework. (a) The first layer CNN model conducts fast localization of BM particles and cellular trails in low resolution as ROI(s), which is then mapped to high magnification level where the second layer network performs BM cell detection and classification inside ROI(s). (b and b2) The region proposal network (RPN) of CW-Net produces candidate bounding boxes using (b3) the proposed soft-sampling weighted loss function to decrease the influence of unlabeled data in AI training and avoid confusions among unlabeled data, targets and background. (b4) Sample classification models are then applied to generate (b5) positive $b^{d+}$ and refined negative samples ${\left(b^{d-}\right)}^{*}$ using (b6) the proposed DL-FNIS to produce better detectors and classifiers in training. In inference, (c) the original SCNMS is replaced with (d) the proposed MCNMS to ensure that there is no contradictory classification

In routine BM examination, examiners first determine an adequate BM smear by the presence and cellularity of particles viewed under low magnification power to avoid diluted regions. Second, examiners perform BM NDC analysis within areas with well spread marrow cells in the cellular trails of the BM smear behind the particles viewed under high magnification power. In this study, we introduce an efficient and fully automatic deep learning method (CW-Net) for multitype cell differentiation of BM NDC WSI analysis in seconds. An overview of the proposed method is shown in Fig. 2. The first layer CNN model rapidly locates BM particles and cellular trails in low resolution as ROI(s), which is used to locate data for further analysis in high magnification level where the second layer CW-Net performs BM cell detection and classification. The base deep learning model of the proposed method is adapted from Cascade R-CNN (Cai and Vasconcelos 2021), which is a multistage object detection and classification method.

#### 2.1.1 Proposed CW-Net architecture

The proposed CW-Net is composed of four components: a backbone, a feature pyramid network (FPN+; Cai and Vasconcelos 2021), a region proposal network (RPN) and a prediction head. Initially, a deep ResNet101 backbone is employed to extract features, and the output of ResNet101 is then sent into FPN+, which integrates features from different levels and creates multiple scale features by up-sampling. The multiscale feature maps created from FPN+ are fed into RPN, which generates proposal bounding boxes and assigns anchors to feature maps of varying sizes. We devised RPN with a Jaccard-based soft sampling weighted loss function, which achieved state-of-the-art performance on partially annotated data in our previous study (Wang *et al.* 2022). In training, soft sampling ensures that all positive and hard negatives will contribute to the gradient, but with a lower attention weight. An illustration of soft-sampling weighting mechanism is given in Fig. 2b3.

**Figure 3.**
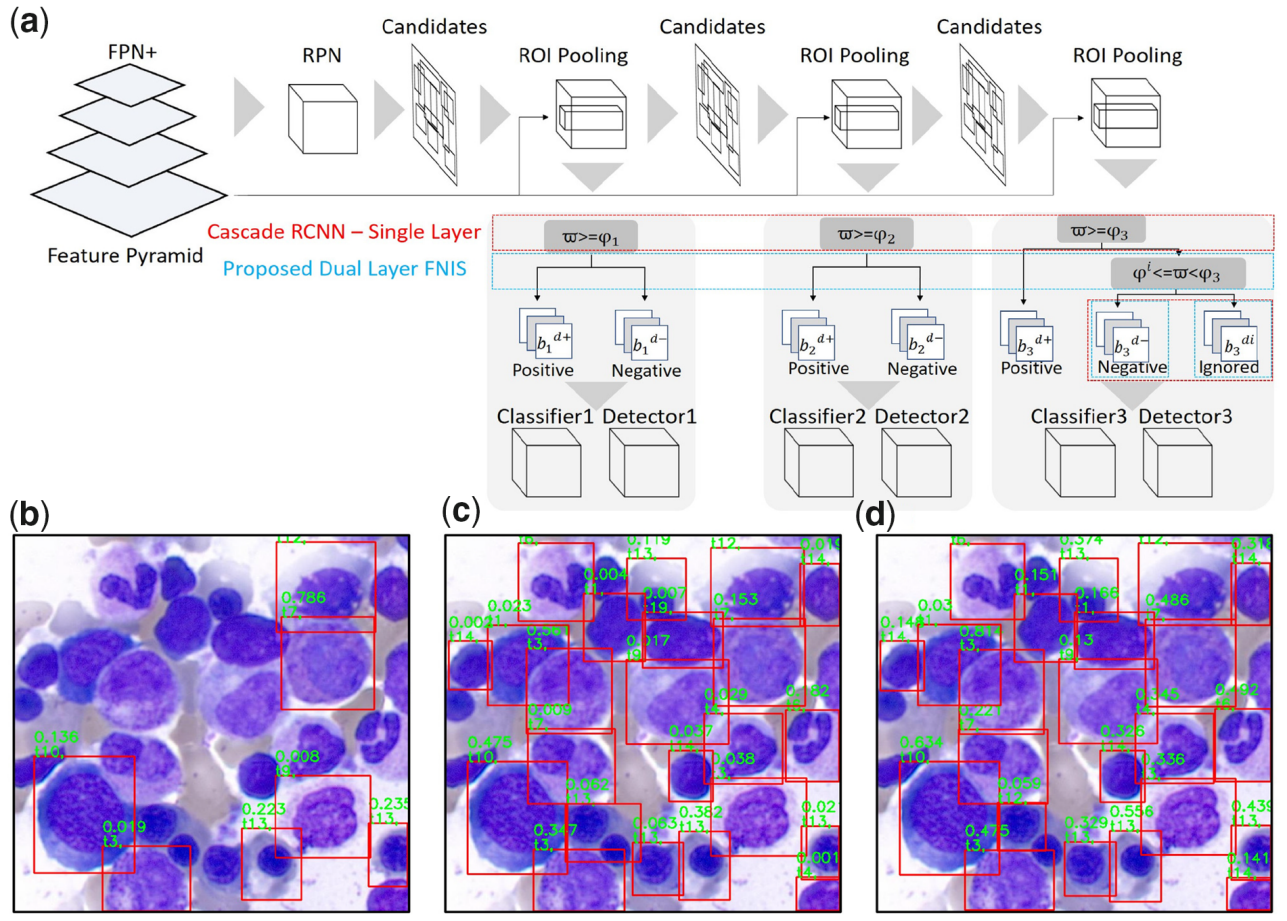
(a) Illustration of the proposed dual layer filtered negative instance sampling (dual layer FNIS). A low threshold 0.001 for the detection-classification confidence of a cell is used for compare (b) the original Cascade R-CNN, (c) the proposed framework without DL-FNIS strategy and (d) the proposed framework with DL-FNIS strategy, showing that even with such a low threshold, for (b) original Cascade R-CNN there are still many cells undetected, while in (c) the proposed framework without DL-FNIS strategy many cells can be detected but with very low confidence rates in classification, which however tend to be filtered out (disqualified) by common criteria such as 0.5. In (d), the proposed framework with DL-FNIS strategy, the number of detected cells is comparably more than (b and c) and the confidence rates of cells are significantly higher. The result shows the effectiveness of DL-FNIS strategy for improving detectors and classifiers in Cascade R-CNN

#### 2.1.2 Dual layer filtered negative instance sampling

As shown in Fig. 3a, single layer binary classification systems are used in Cascade R-CNN to generate positive samples $b^{d+}$ and negative samples $b^{d-}$ from candidate bounding boxes $b^{d}$ to train multiple classifiers with thresholds $\varphi ={\left\{\varphi_{z}\right\}}_{z=1\ldots Z}$. where $\varphi_{1}=0.5,\rho =0.1$ is the increasing confidence factor; *Z* is the number of classifiers, and *Z *=* *3 in this study.


(1) $$\varphi_{z+1}=\varphi_{z}+\rho$$

(2) $$b_{z}^{d}=\{\begin{array}{cc} b_{z}^{d+}, & \varpi \geq \varphi_{z},\\ b_{z}^{d-}, & \text{otherwise} \end{array}$$

However, for partially labeled data, the single layer design causes serious confusions in AI learning and downgrades the resulting detectors and classifiers because a number of unlabeled cells will be used as negative samples for training. As a result, to resolve this issue caused by partial annotations, we propose a dual layer filtered negative instance sampling (DL-FNIS) strategy by adding an extra layer in selection of positive and negative samples with a new sample type, i.e. the ignored class $b_{Z}^{di}$, in consideration with the Jaccard index ϖ between the reference standards and the candidate bounding boxes to the last stage model in sample classification as shown in Fig. 3a, producing positive samples $b_{Z}^{d+}$ and refined negative samples ${\left(b_{Z}^{d-}\right)}^{*}$ for training *Z*th classifier and detector. The second layer binary classification system of the proposed DL-FNIS is devised to select refined negative samples for training and is formulated as follows. where $\varphi^{i}=0.1$ is used to define ignored instances for training in this study.


(3) $$b_{Z}^{d-}=\{\begin{array}{cc} {\left(b_{Z}^{d-}\right)}^{*}, & \varpi \geq \varphi^{i},\\ b_{Z}^{di}, & \text{otherwise} \end{array}$$

To further illustrate the contributions of DL-FNIS, Fig. 3b compare the outputs of the original Cascade R-CNN, the proposed CW-Net without DL-FNIS and the proposed CW-Net with DL-FNIS using a low threshold for detection-classification confidence of a cell, showing that even with such a low threshold, for (b) original Cascade R-CNN there are still many cells undetected, while for (c) the proposed CW-Net without DL-FNIS many cells can be detected but with very low confidence rates in classification, which however tend to be filtered out (disqualified) by common criteria such as 0.5, and in (d) the proposed CW-Net with DL-FNIS, the number of detected cells is comparably more than (b and c) and confidence rates of cells are significantly higher. The result shows the effectiveness of DL-FNIS for improving both detectors and classifiers.

#### 2.1.3 Multiclass nonmaximum suppression

In the original Cascade R-CNN design, it was found that a single cell object may be misidentified as various kinds of cells because single class nonmaximum suppression (SCNMS) is utilized for inference as shown in Fig. 2c. For each class, the initial output set $o_{c}$ are rendered if the probability of a detected object is greater than ϱ. where $p_{\left(c_{b^{d}},b^{d}\right)}$ is detected BM cell type probability, ϱ is the classification threshold and ϱ=0.5 in this study.


(4) $$o_{c}={\left\{(c_{b^{d\prime }},b^{d\prime }){|}_{p_{\left(c_{b^{d}},b^{d}\right)}\geq \varrho }\right\}}_{d=1}^{D\prime <D}$$

SCNMS aims to suppress the initial detection results $o_{c}$ at each class to generate the SCNMS output $O_{\mathrm{scnms}}$. where ϖ′ is the Jaccard index between the detected BM cells (*b^i^*, *b^j^*), and *η *= 0.3 in this study.


(5) $$(c_{b^{d^{*}}},b^{d^{*}})=\underset{i}{\text{argmax}}(p_{\left(c_{b^{i}},b^{i}\right)}){|}_{\varpi \prime \geq \eta \;\wedge \;c_{b^{i}}=c_{b^{j}}}$$

(6) $$O_{\mathrm{scnms}}=\overset{K}{\underset{c=1}{\cup }}\left(c_{b^{d^{*}}},b^{d^{*}}\right)$$

In inference, SCNMS is replaced with a MCNMS strategy, which ensure no contradictory prediction of a cell. This strategy greedily selects a subset of detection bounding boxes by pruning away boxes that have high Jaccard overlap with already selected boxes.

MCNMS produces the output set $O_{\mathrm{MCNMS}}$ with all class at once as formulated as follows. where ϖ′ is the Jaccard index between the detected BM cells (*b^i^*, *b^j^*), and *η *= 0.3 in this study.


(7) $$\left(c_{b^{d^{**}}},b^{d^{**}})=\underset{i}{\text{argmax}}(p_{\left(c_{b^{i}},b^{i}\right)}{|}_{\varpi \prime \geq \eta }\right)$$

(8) $$O_{\mathrm{mcnms}}=\overset{K}{\underset{c=1}{\cup }}\left(c_{b^{d^{**}}},b^{d^{**}}\right)$$

#### 2.1.4 Data augmentation and normalization

Methods trained with images from one hospital tend to perform poorly on images from other hospitals, even for state-of-the-art deep learning-based methods (Tellez *et al.* 2019). Minimizing generalization error is important for building a robust AI model for unseen data. Data augmentation could operate as a regularizer in neural networks, minimizing overfitting and improving performance when dealing with unbalanced classes. During training, data augmentation mimics a broad range of actual changes, generating CNNs robust to variations in stain, translation, perspective, size or lighting. Data normalization, on the other hand, is designed to reduce data variation and therefore improving model generalizability. In this work, we built a Jaccard-based data augmentation method and a data normalization process for reducing the model generalization error. First, the data augmentation is applied to the selected patches if the associated Jaccard coefficient $\eta_{z_{k}}$, which is determined in Equation (9), of an individual patch is >0. The selected patches are used to supplement the training set with new synthetically changed data with the following operations, including rotation per 5^∘^ and 5 times and increment of 90^∘^, the mirror-flipped along the horizontal and vertical axes, the contrast adjusted (random contrast, range 0%±20%), the saturation adjusted (random saturation, range 0%±20%) and the brightness adjusted (random brightness, range 0%±12.5%) during the training process. where ***h*** is the reference positive samples, and *z_k_* is a patch. Second, a data normalization method is built to maintain data consistency and gradient stability in the training dataset, as well as to prevent overflow from data augmentation. The data normalization is performed by histogram specification, which matches the color distribution *P_S_* of input data *S* to a reference distribution *P_R_* trained from the ImageNet dataset (Deng *et al.* 2009) and produces a normalized data S′.


(9) $$\eta_{z_{k}}=\frac{h\cap z_{k}}{z_{k}}$$

#### 2.1.5 Adaptive learning

Cascade R-CNN uses a fixed step size for reducing the learning rate by 10% at 160k and 240k iterations. However, the training instances contribute less to the learned model as the learning rate decreases, and some instances may not be learned adequately if the learning rate reduces dramatically. To address this problem, we proposed an adaptive learning (AL) technique with a flexible data-oriented learning rate adjustment mechanism (ϱ). Given the number of training images *I*, the size of each image *w *×* h*, the size of each unit patch *q *×* q*, the AL rate $r_{\Lambda }$ at iteration Λ is formulated as follows: where $I\times \frac{w}{q}\times \frac{h}{q}$ represents the total number of patches in the training data, excluding data augmentation, and α=10%.


(10) $$r_{\Lambda }=1-\left\lfloor \frac{\Lambda }{\varrho }\right\rfloor \times \alpha$$

(11) $$\varrho =\frac{I}{\alpha }\times \frac{w}{q}\times \frac{h}{q}$$

## 3 Experiments and results

### 3.1 Quantitative evaluation

#### 3.1.1 Identification of 16 types of bone marrow cells

**Figure 4.**
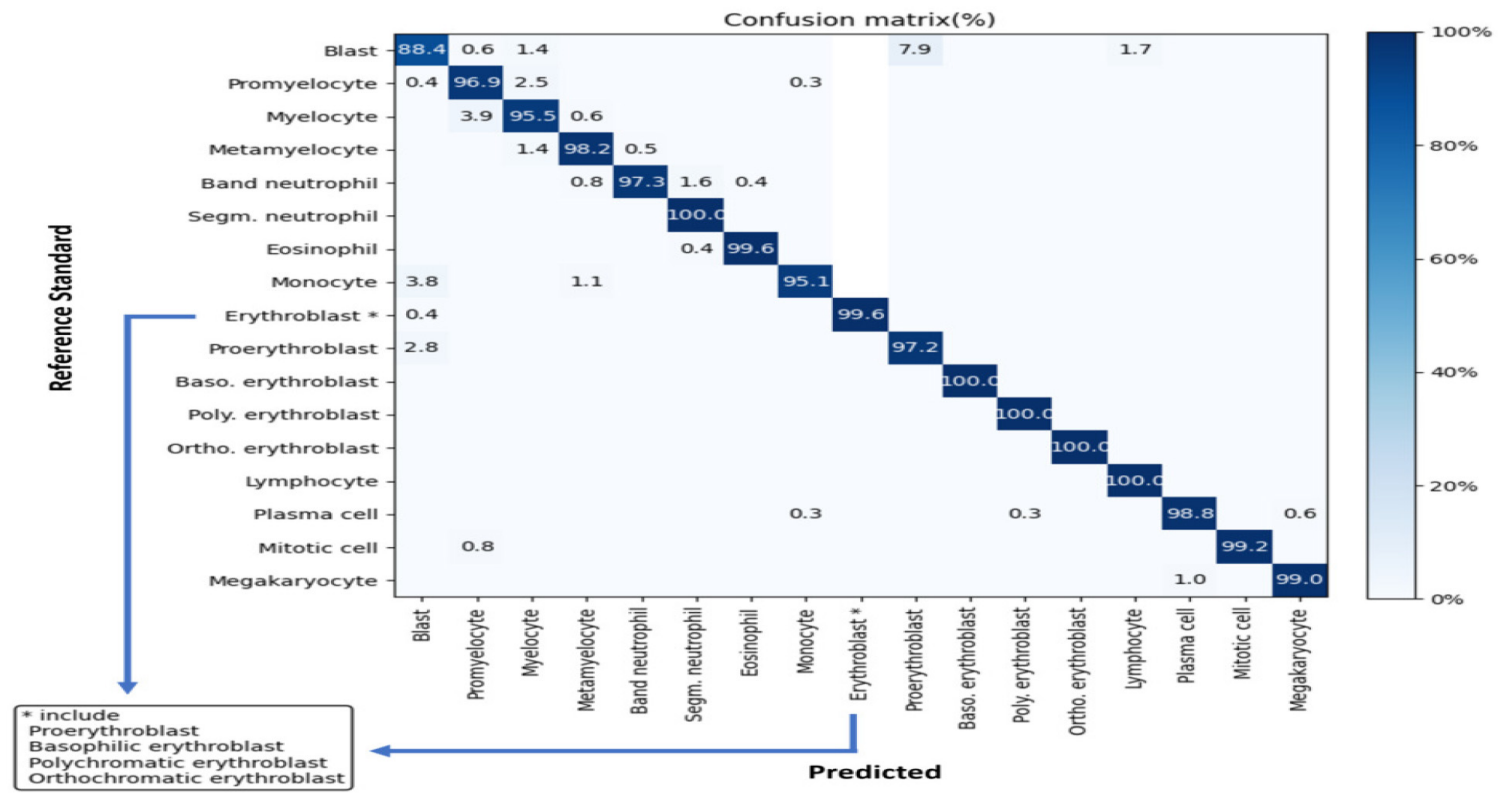
Confusion matrix for 17 cell types (in percentage) by comparing the output of the proposed method and the reference standard of validation dataset

**Figure 5.**
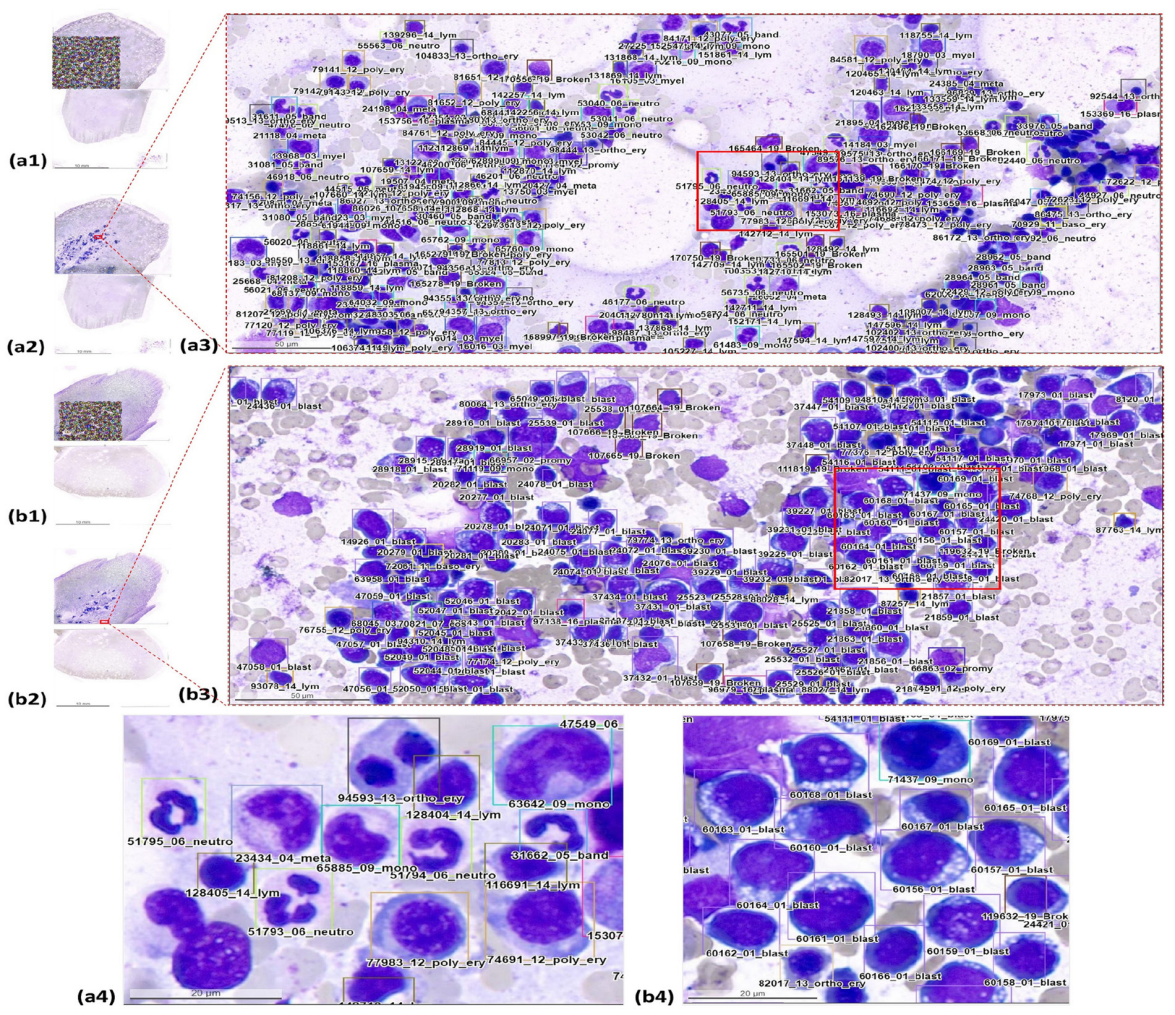
Results of BM NDC analysis by the proposed method on (a) a normal WSI sample and (b) a diseased WSI sample with acute lymphoblastic leukemia (ALL). (1) A global view of the WSI with detection results, (2) a global view of the WSI with the location of (3) the medium zoom-in view and (4) a high-resolution zoom-in view

Quantitative evaluation was performed with patch-wise 10-fold cross validation, for which cells on the same patches were used exclusively in 1-fold and never assigned to both training and testing set at the same time, and the proposed CW-Net was compared with three recently published benchmark approaches, including two small-image-based approaches (Yu *et al.* 2019, Chandradevan *et al.* 2020) and our previous work for BM NDC WSI analysis (Wang *et al.* 2022; see Table 1 for the results where the best results are highlighted in bold case and the reported numbers of Yu *et al.* 2019, Chandradevan *et al.* 2020, Wang *et al.* 2022 are referred). The experimental results show that the proposed CW-Net demonstrates superior performance in BM NDC analysis in WSIs with an averaged recall of 0.974 ± 0.032, an averaged accuracy of 0.997 ± 0.003 and an averaged PR-AUC of 0.985 ± 0.027. Moreover, the proposed method consistently achieves >0.99 accuracy for all BM cell types and >0.95 recall for most types, respectively. In comparison with two recent BM NDC analysis methods (Yu *et al.* 2019, Chandradevan *et al.* 2020), which require human intervention to manually cropped small image areas, the proposed fully automatic CW-Net method outperforms both benchmark approaches for identification of all cell types. Moreover, in comparison with Cascade R-CNN, the proposed CW-Net consistently obtains higher recalls and accuracies, and importantly the proposed CW-Net achieves high recall values in identification of many cell types such as blast, promyelocyte, myelocyte and monocyte, which are comparably low by Cascade R-CNN and notably boosted up >10%–37%. Furthermore, based on confusion matrix provided in Fig. 4, the proposed method shows excellent performance for 16 of the 17 BM cells. To sum up, the proposed CW-Net greatly improves the AI model performance, and the proposed framework could also be integrated to other CNN methods to improve model performance. Figure 5 presents sample results by the proposed method, and a discussion on the system limitation is provided in Supplementary Section S3.

**Table 1.** Quantitative evaluation in identification of BM cells.

Method	Chandradevan *et al.* (2020) ^a^		Yu *et al.* (2019) ^a^		Wang *et al.* (2022) ^a^		Proposed cascaded framework					
Base model							Cascade R-CNN (Cai and Vasconcelos 2021)		Proposed CW-Net		CW-Net without FNIS	
Human intervention	Manual cropped		Manual cropped		Fully automatic		Fully automatic		Fully automatic		Fully automatic	
Data type	Small images		Small images		WSIs		WSIs		WSIs		WSIs	
Cell type	Recall	Accuracy	Recall	Accuracy	Recall	Accuracy	Recall	Accuracy	Recall	Accuracy	Recall	Accuracy
Blast	0.850	0.974	0.815	0.956	**0.974 ± 0.022**	0.978 ± 0.009	0.764 ± 0.350	0.968 ± 0.014	0.884 ± 0.136	**0.994 ± 0.005**	0.792 ± 0.332	**0.996 ± 0.005**
Promyelocyte	0.870	0.963	0.854	0.984	0.877 ± 0.049	0.976 ± 0.008	0.767 ± 0.212	0.963 ± 0.019	**0.969 ± 0.028**	**0.994 ± 0.004**	**0.993 ± 0.015**	**0.998 ± 0.002**
Myelocyte	0.820	0.972	0.708	0.969	0.757 ± 0.112	0.982 ± 0.008	0.675 ± 0.266	0.972 ± 0.015	**0.955 ± 0.040**	**0.993 ± 0.005**	**0.975 ± 0.042**	**0.997 ± 0.003**
Metamyelocyte	0.840	0.971	0.847	0.968	0.876 ± 0.075	0.986 ± 0.006	0.866 ± 0.256	0.987 ± 0.013	**0.982 ± 0.044**	**0.998 ± 0.003**	**0.981 ± 0.044**	**0.998 ± 0.003**
Bandneutrophil	0.900	0.988	0.876	0.974	0.852 ± 0.147	0.991 ± 0.006	0.876 ± 0.200	0.988 ± 0.010	**0.973 ± 0.046**	**0.998 ± 0.003**	**0.986 ± 0.030**	**0.998 ± 0.002**
Segmentedneutrophil					0.946 ± 0.057	0.991 ± 0.005	0.957 ± 0.047	0.989 ± 0.010	**1 ± 0**	**0.998 ± 0.002**	**1 ± 0**	**0.999 ± 0.001**
Eosinophil	0.940	0.993	0.987	0.990	0.923 ± 0.159	0.999 ± 0.003	0.946 ± 0.089	0.998 ± 0.003	**0.996 ± 0.014**	**0.999 ± 0.001**	**1 ± 0**	**1 ± 0**
Monocyte	0.690	0.973	0.825	0.982	0.651 ± 0.284	0.990 ± 0.008	0.580 ± 0.389	0.987 ± 0.013	**0.951 ± 0.093**	**0.998 ± 0.002**	**0.971 ± 0.067**	**0.998 ± 0.002**
Erythroblast	0.860	0.979	0.971	0.984	0.980 ± 0.020	0.979 ± 0.020	0.988 ± 0.029	0.988 ± 0.003	**0.996 ± 0.009**	**0.996 ± 0.009**	**0.999 ± 0.004**	**0.999 ± 0.004**
Proerythroblast					0.900 ± 0.108	0.996 ± 0.004	0.930 ± 0.03	0.995 ± 0.004	**0.972 ± 0.062**	**0.996 ± 0.005**	**1 ± 0**	**0.997 ± 0.005**
Baso.erythroblast					0.892 ± 0.013	0.983 ± 0.005	**1 ± 0**	0.983 ± 0.005	**1 ± 0**	**1 ± 0**	**0.900 ± 0.316**	**1 ± 0**
Poly.erythroblast					0.996 ± 0.023	0.973 ± 0.012	0.997 ± 0.010	0.973 ± 0.012	**1 ± 0**	**0.999 ± 0.001**	**0.997 ± 0.010**	**0.999 ± 0.001**
Ortho.erythroblast					0.992 ± 0.088	0.984 ± 0.010	0.943 ± 0.295	0.984 ± 0.010	**1 ± 0**	**1 ± 0**	**1 ± 0**	**1 ± 0**
Lymphocyte	0.680	0.970	0.750	0.927	0.945 ± 0.093	0.996 ± 0.002	0.937 ± 0.100	0.992 ± 0.030	**1 ± 0**	**1 ± 0**	**1 ± 0**	**0.999 ± 0.002**
Plasma cell	0.890	0.990			0.987 ± 0.015	0.997 ± 0.002	**0.995 ± 0.014**	0.994 ± 0.008	0.988 ± 0.016	**0.998 ± 0.002**	0.987 ± 0.017	**0.998 ± 0.002**
Mitotic cell					**1 ± 0**	**1 ± 0.001**	0.975 ± 0.070	0.999 ± 0.002	**0.992 ± 0.026**	**1 ± 0**	**1 ± 0**	**1 ± 0**
Megakaryocyte					0.997 ± 0.009	**1 ± 0.001**	**1 ± 0**	**1 ± 0**	0.990 ± 0.032	0.999 ± 0.002	0.990 ± 0.032	0.999 ± 0.002
Basophil	0.890	0.988	0.818	0.987								
Invalid/unknown	0.920	0.928	0.666	0.877								
Average	0.846	0.974	0.829	0.964	0.905 ± 0.078	0.989 ± 0.006	0.848 ± 0.200	0.989 ± 0.006	**0.974 ± 0.032**	**0.997 ± 0.003**	**0.973 ± 0.055**	**0.998 ± 0.002**
PR-AUC	0.959						0.901 ± 0.168		**0.985 ± 0.027**		**0.988 ± 0.023**	

a The reported numbers from Chandradevan *et al.* (2020), Yu *et al.* (2019) and Wang *et al.* (2022) are referred in this table.

#### 3.1.2 Intra- and interobserver reliability analysis

Cohen’s kappa statistic is used to analyze the annotation agreement of intra- and interobserver. The intraobserver analysis is performed based on two sets of annotations produced at an interval of one week. For the intraobserver variability, we perform the kappa analysis on 665 randomly selected BM cells from three WSIs, and for the interobserver variability, we perform the kappa analysis on 1966 randomly selected BM cells from five WSIs (see Supplementary Table S1). Conventionally, a kappa value of <0.20, 0.21–0.40, 0.41–0.60, 0.61–0.80 and 0.81–1.00 are interpreted as a poor, fair, moderate, good and excellent agreement, respectively (Gianelli *et al.* 2014). The intraobserver reliabilities of examiner 1 and 2 are interpreted as good with kappa values of 0.608 and 0.789, respectively, and the interobserver reliability between the two examiners is also good with a kappa value of 0.8.

For the interobserver analysis between AI and examiners, high kappa values of 0.824 and 0.908 were obtained, showing that the proposed AI model is reliable and highly consistent to the specialized medical examiners’ decisions. Moreover, the results show that the second examiner who has >20 years of expertise in BM NDC analysis produces more consistent decisions, obtaining higher intraobserver kappa than the first examiner. In addition, the results of interobserver analysis show that the mean kappa between the proposed AI model and the senior examiner is higher than the one with the junior examiner. More information is provided in Supplementary Section S4.

#### 3.1.3 Mitotic figure examination of five cell types

Quantitative results are presented to evaluate the robustness and effectiveness of the proposed CW-Net method in identification of mitotic cells, with comparison of eight recently published state-of-the-art approaches, including the Top 3 methods in 2021 challenge, in Tables 2 and 3 where the best results are highlighted in bold cases, and the reported numbers of Li *et al.* (2018), Bertram *et al.* (2019), Alom *et al.* (2020), Cai *et al.* (2021), Sohail *et al.* (2021), Aubreville *et al.* (2023) are referred. Table 2 shows that the proposed CW-Net demonstrates superior performance than the benchmark methods in identification of mitotic figures with a recall of 0.843, precision of 0.858 and f1-score of 0.851 in detection and high precision of 0.841, recall of 0.876 in classification of mitotic figure, respectively. Furthermore, the proposed CW-Net without FNIS achieves the second best precision of 0.762 and f1-score of 0.761 in detection and the highest recall of 0.943 and the second best f1-score of 0.862 in classification of mitotic figure, respectively.

**Table 2.** Quantitative evaluation in mitotic figure examination: comparison of benchmark methods and the proposed methods in identification of mitotic figure.

Data type	WSI					
	Detection			Classification		
Method	Recall	Precision	F1-score	Recall	Precision	F1-score
DeepMitotis^a^ (Li *et al.* 2018)	0.443	0.431	0.437			
Bertram *et al.*^a^ (Bertram *et al.* 2019)	0.688	0.577	0.628	0.812	0.828	0.820
Modified R-CNN^a^ (Cai *et al.* 2019)	0.720	0.760	0.736			
MitosisNet^a^ (Alom *et al.* 2020)	0.782	0.740	0.759			
MP-MitDet^a^ (Sohail *et al.* 2021)	0.768	0.734	0.75			
2021 Challenge#1 (AI medical)^b^			0.748			
2021 Challenge#2 (TIA Centre)^b^			0.747			
2021 Challenge#3 (Tribvn Healthcare)^b^			0.736			
Proposed CW-Net	**0.843**	**0.858**	**0.851**	0.914	**0.841**	**0.876**
Proposed CW-Net without FNIS	0.762	0.761	0.761	**0.943**	0.749	0.862

a The reported numbers from Li *et al.* (2018), Bertram *et al.* (2019), Cai *et al*. (2019), Alom *et al.* (2020) and Sohail *et al.* (2021) are referred in this table.

b Results of the Top 3 teams in 2021 MIDOG Challenge (Aubreville *et al.* 2023) held with the MICCAI conference are referred.

**Table 3.** Quantitative evaluation in mitotic figure examination: comparison of the proposed methods in classification of multitype mitotic cells.

	Proposed CW-Net			Proposed CW-Net without FNIS		
Data type	WSI			WSI		
Cell type	Recall	Precision	F1-score	Recall	Precision	F1-score
Granulocyte	**0.995**	**0.978**	**0.986**	**0.903**	**0.893**	**0.897**
Mitotic figure	**0.914**	**0.841**	**0.876**	**0.943**	**0.749**	**0.862**
Tumor cells	**0.990**	**0.994**	**0.992**	**0.961**	**1.0**	**0.979**
Mitotic figure lookalike	0.567	0.670	0.614	0.469	0.740	0.563
Other ambiguous cells	0.258	0.957	0.407	0.267	0.975	0.330

**Figure 6.**
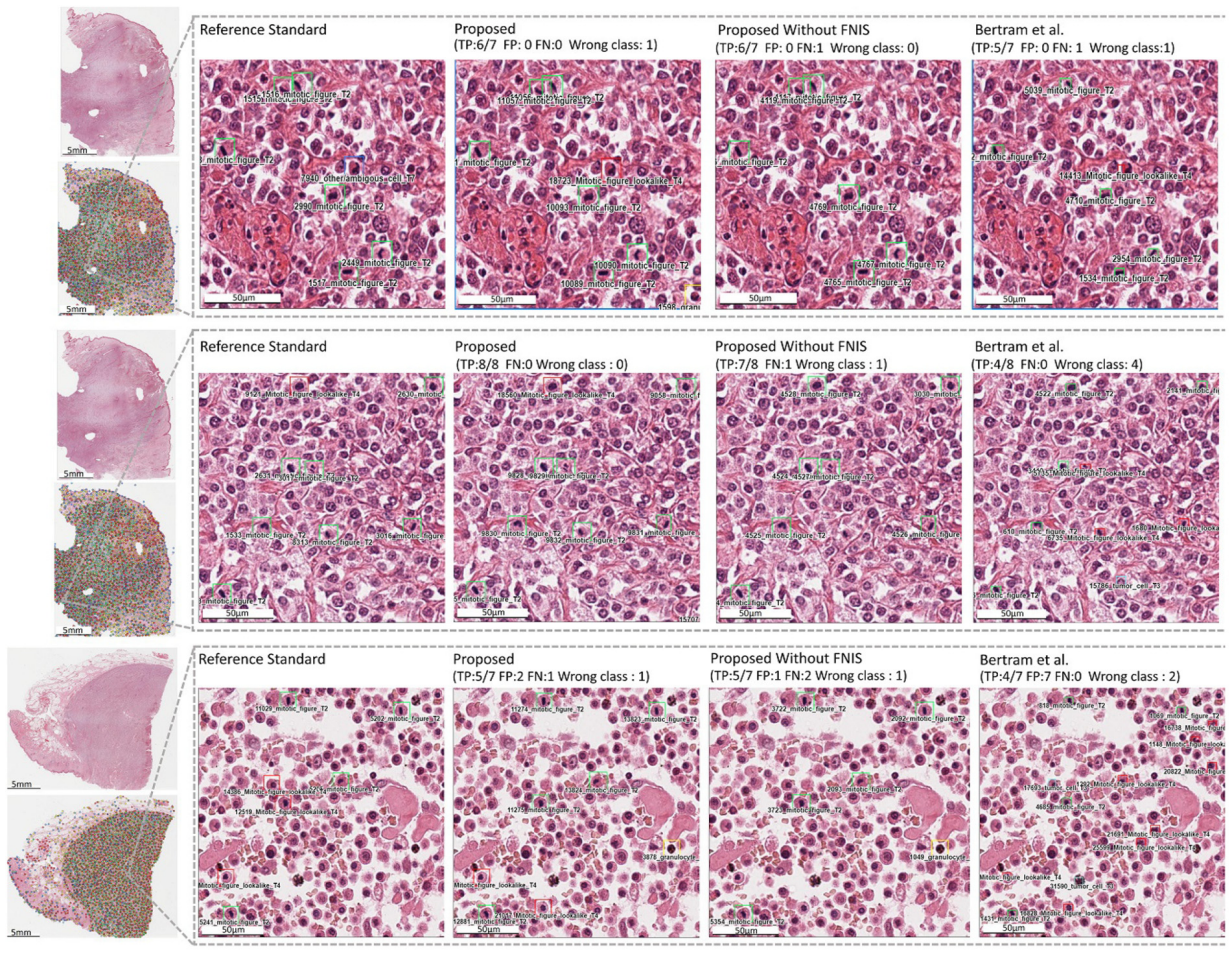
Sample results of mitotic analysis by the proposed CW-Net and one of the benchmark method (Bertram *et al.* 2019)

Table 3 further compares the performance of the proposed CW-Net with and without FNIS strategy in classification of five types of mitotic cells, including granulocyte, mitotic figure, tumor cells, mitotic figure lookalike and other ambiguous cells. The experimental results indicate that the proposed CW-Net achieves excellent performance in classification of granulocyte, mitotic figure and tumor cells. The sample results of mitotic analysis by the proposed CW-Net and one of the benchmark method (Bertram *et al.* 2019) are displayed in Fig. 6.

## 4 Conclusion

Fully automated examinations of BM slides and mitotic figures are highly demanded but challenging. First, the complexity and poor reproducibility of BM and mitotic figure examination on WSIs emerge from the cell type diversity, delicate intralineage difference within the maturation process of multitype cells, cell overlapping, lipid interference and stain variations. Second, manual annotation on WSIs with enormous data dimensions and complicated cell types is difficult, which causes the supervised information restricted to limited, easily identifiable and scattered cells annotated by human. In this article, we develop a fully automatic and efficient cascaded weakly supervised deep learning framework (CW-Net) for multitype cell detection and classification for both BM examination and mitotic figure examination. Comprehensive experiments demonstrate that the proposed method has the discriminative ability in both applications and achieves state-of-the-art performance.

## Supplementary Material

btad344_Supplementary_Data

## Data Availability

The data underlying this article cannot be shared publicly due to the requirement of the ethical approval. The data will be shared on reasonable request to the corresponding author.

## References

[btad344-B1] Alom MZ , Aspiras T, Taha TM et al Mitosisnet: end-to-end mitotic cell detection by multi-task learning. IEEE Access 2020;8:68695–710.

[btad344-B2] Aubreville M , Stathonikos N, Bertram CA et al Mitosis domain generalization in histopathology images—the MIDOG challenge. Med Image Anal 2023;84:102699. 36463832 10.1016/j.media.2022.102699

[btad344-B3] Balkenhol MC , Tellez D, Vreuls W et al Deep learning assisted mitotic counting for breast cancer. Lab Invest 2019;99:1596–606. 31222166 10.1038/s41374-019-0275-0

[btad344-B4] Bertram CA , Aubreville M, Marzahl C et al A large-scale dataset for mitotic figure assessment on whole slide images of canine cutaneous mast cell tumor. Sci Data 2019;6:1–9. 31754105 10.1038/s41597-019-0290-4 PMC6872565

[btad344-B5] Bertram CA , Aubreville M, Gurtner C et al Computerized calculation of mitotic count distribution in canine cutaneous mast cell tumor sections: mitotic count is area dependent. Vet Pathol 2020;57:214–26. 31808382 10.1177/0300985819890686

[btad344-B6] Bray F , Ferlay J, Soerjomataram I et al Global cancer statistics 2018: Globocan estimates of incidence and mortality worldwide for 36 cancers in 185 countries. CA Cancer J Clin 2018;68:394–424. 30207593 10.3322/caac.21492

[btad344-B7] Cai D , Sun X, Zhou N et al Efficient mitosis detection in breast cancer histology images by R-CNN. In: *2019 IEEE 16th International Symposium on Biomedical Imaging (ISBI 2019), Venice, Italy, 08-11 April 2019*, 919–22. IEEE, 2019.

[btad344-B8] Cai Z , Vasconcelos N. Cascade R-CNN: high quality object detection and instance segmentation. IEEE Trans Pattern Anal Mach Intell 2021;43:1483–98. 10.1109/TPAMI.2019.2956516. 31794388

[btad344-B9] Campanella G , Hanna MG, Geneslaw L et al Clinical-grade computational pathology using weakly supervised deep learning on whole slide images. Nat Med 2019;25:1301–9. 31308507 10.1038/s41591-019-0508-1 PMC7418463

[btad344-B10] Chandradevan R , Aljudi A, Drumheller B et al Machine-based detection and classification for bone marrow aspirate differential counts: initial development focusing on nonneoplastic cells. Lab Invest 2020;100:98–109. 10.1038/s41374-019-0325-7. 31570774 PMC6920560

[btad344-B11] Deng J , Dong W, Socher R et al Imagenet: a large-scale hierarchical image database. In: *2009 IEEE Conference on Computer Vision and Pattern Recognition, Miami, FL, USA*, 248–55, 2009. 10.1109/CVPR.2009.5206848.

[btad344-B12] Gianelli U , Bossi A, Cortinovis I et al Reproducibility of the who histological criteria for the diagnosis of Philadelphia chromosome-negative myeloproliferative neoplasms. Mod Pathol 2014;27:814–22. 24201120 10.1038/modpathol.2013.196

[btad344-B13] Greenberg PL , Tuechler H, Schanz J et al Revised international prognostic scoring system for myelodysplastic syndromes. Blood 2012;120:2454–65. 10.1182/blood-2012-03-420489. 22740453 PMC4425443

[btad344-B14] Kumar S , Paiva B, Anderson KC et al International myeloma working group consensus criteria for response and minimal residual disease assessment in multiple myeloma. Lancet Oncol 2016;17:e328–46. 27511158 10.1016/S1470-2045(16)30206-6

[btad344-B15] Lee S-H , Erber W, Porwit A, and I. C. S. I. Hematology et al ICSH guidelines for the standardization of bone marrow specimens and reports. Int J Lab Hematol 2008;30:349–64. 18822060 10.1111/j.1751-553X.2008.01100.x

[btad344-B16] Li C , Wang X, Liu W et al Deepmitosis: mitosis detection via deep detection, verification and segmentation networks. Med Image Anal 2018;45:121–33. 29455111 10.1016/j.media.2017.12.002

[btad344-B17] Ludovic R , Daniel R, Nicolas L et al Mitosis detection in breast cancer histological images an ICPR 2012 contest. J Pathol Inform 2013;4:8. 23858383 10.4103/2153-3539.112693 PMC3709417

[btad344-B18] Malon C , Brachtel E, Cosatto E et al Mitotic figure recognition: agreement among pathologists and computerized detector. Anal Cell Pathol 2012;35:97–100. 10.3233/ACP-2011-0029 PMC4605539 21965283

[btad344-B19] Meintker L , Ringwald J, Rauh M et al Comparison of automated differential blood cell counts from Abbott Sapphire, Siemens Advia 120, Beckman Coulter DxH 800, and Sysmex XE-2100 in normal and pathologic samples. Am J Clin Pathol 2013;139:641–50. 23596116 10.1309/AJCP7D8ECZRXGWCG

[btad344-B20] Roux L. Detection of mitosis and evaluation of nuclear atypia score in breast cancer histological images. In: *22nd International Conference on Pattern Recognition, Stockholm, Sweden*, 2014.

[btad344-B21] Sohail A , Khan A, Wahab N et al A multi-phase deep CNN based mitosis detection framework for breast cancer histopathological images. Sci Rep 2021;11:1–18. 33737632 10.1038/s41598-021-85652-1 PMC7973714

[btad344-B22] Swerdlow S , Campo E, Harris NL et al *WHO Classification of Tumours of Haematopoietic and Lymphoid Tissues*, revised 4th edn. Lyon: IARC, 2017, p.421.

[btad344-B23] Tellez D , Litjens G, Bándi P et al Quantifying the effects of data augmentation and stain color normalization in convolutional neural networks for computational pathology. Med Image Anal 2019;58:101544. 31466046 10.1016/j.media.2019.101544

[btad344-B24] Veta M , Heng YJ, Stathonikos N et al Predicting breast tumor proliferation from whole-slide images: the tupac16 challenge. Med Image Anal 2019;54:111–21. 30861443 10.1016/j.media.2019.02.012

[btad344-B25] Wang C-W , Huang S-C, Lee Y-C et al Deep learning for bone marrow cell detection and classification on whole-slide images. Med Image Anal 2022;75:102270. 34710655 10.1016/j.media.2021.102270

[btad344-B26] Yu T-C , Chou W-C, Yeh C-Y et al Automatic bone marrow cell identification and classification by deep neural network. Blood 2019;134:2084. 10.1182/blood-2019-125322.

