# Supplementary material for: CW-NET for multitype cell detection and classification in bone marrow examination and mitotic figure examination
Source: Bioinformatics. 2023 May 30;39(6):btad344. doi: 10.1093/bioinformatics/btad344 (PMC10243868; doi:10.1093/bioinformatics/btad344)
Supplement: btad344_Supplementary_Data [file btad344_supplementary_data.pdf]

Supplementary Information

CW-NET for Multi-type Cell Detection and Classification in Bone Marrow Examination and Mitotic Figure Examination

Ching-Wei Wang,[1,2,*] Sheng-Chuan Huang,[3,4,5] Muhammad-Adil Khalil,[1] Ding-Zhi Hong,[2] Shwu-Ing Meng,[3] Yu-Ching Lee[1]

[1] Graduate Institute of Applied Science and Technology, National Taiwan University of Science and Technology, Taipei, Taiwan.
[2] Graduate Institute of Biomedical Engineering, National Taiwan University of Science and Technology, Taipei, Taiwan.
[3] Department of Laboratory Medicine, National Taiwan University Hospital, Taipei, Taiwan.
[4] Department of Hematology and Oncology, Hualien Tzu Chi Hospital, Buddhist Tzu Chi Medical Foundation, Hualien, Taiwan.
[5] Department of Clinical Pathology, Hualien Tzu Chi Hospital, Buddhist Tzu Chi Medical Foundation, Hualien, Taiwan.

[*] Corresponding author: Ching-Wei Wang. Email: cweiwang@mail.ntust.edu.tw

S1: Related Works

S1.1: Weakly Supervised Method

Weakly supervised object detection (WSOD) has been demonstrated to be useful in applications with a few fully-annotated images (supervision) to detect a large number of non-fully-annotated images, and traditionally models are learned from images labeled with the object class only, not the bounding box [15]. WSOD has also been utilized for many medical image analysis applications. Hu et al. [14] proposed a novel framework for image registration on MR images. Wang et al. [23] developed a weakly supervised deep learning framework using 3D CT volumes for COVID-19 classification and lesion detection. Bilen et al [4] presented a representative framework for weakly supervised object detection, which only needs fine-tuning on a target data set using back-propagation, region proposals and image-level labels. Tang et al. [25] built a framework to refine a classifier and to re-score proposal bounding boxes to get more discriminative performance of a classifier. Cao et al. [6] designed a Feedback CNN, consisting of the pruning and recovering operations, to capture high-level semantic concepts and transform them into the image space to generate the energy maps that can be enhanced to accurately localize and segment the objects of interest in images.

S1.2: (Semi)-automatic Bone Marrow Analysis Method

Several studies have been conducted to aid BM NDC analysis on simple single cell image patches, acquired by manual selection, utilizing image processing, standard machine learning or deep learning methods. Reta *et al.* [19] presented a contextual method to examine bone marrow cell images to segment and classify five sub-types of acute leukemia. Ghosh *et al.* [11] developed a method that examines the shape, nucleus size, cytoplasm color and textural properties to discriminate six BM cell types on single cell image patch. Mishra *et al.* [18] integrated watershed segmentation, probabilistic principal component analysis and random forest to discriminate the BM cells as benign or malignant. Choi *et al.* [9] introduced a dual-stage deep learning framework to classify BM smear images into 10 myeloid and erythroid maturation series cell types. Kimura *et al.* [16] built a deep learning framework to categorize BM cells into 17 BM cell types and used Extreme Gradient Boosting for further classification of samples into MDS or aplastic anemia (AA). Sahlol *et al.* [20] combined CNN with a statistically enhanced Salp Swarm Algorithm to discriminate the BM cells as benign or malignant.

Recently, there has been improvements in employing multiple-cell images for BM NDC analysis, but most studies are still limited to small manually cropped image patches instead of WSIs. Xie *et al.* [24] employed a fully residual CNN for BM cell detection. Chandradevan *et al.* [7] proposed a semi-automatic method for detecting and classifying 13 different types of BM cells on manually cropped regions. Hu *et al.* [13] used Generative Adversarial Networks [12] for nucleus segmentation and cell classification tasks that include no more than four types

of BM cells. Yu *et al.* [26] adopted a deep learning model to classify BM cells into 11 BM cell types. In our previous work [22], we introduced the first fully automatic method for BM NDC analysis on WSIs. In this study, we present an extended and improved CW-Net deep learning model that achieves better performance than the previous effort [22] with a much larger WSI dataset. As automatic BM NDC WSI analysis is poorly explored, the two recently published small-image-based approaches [7, 26] and our previous work [22] are adopted as benchmark methods.

S1.3: (Semi)-automatic Mitotic Figure Assessment Method

Due to significant advances in computer power and image-scanning technology in recent years, researchers have utilized machine learning or deep learning techniques for mitotic figure detection. Chen et al. [8] developed a two stage framework (CasNN) for mitotic cell detection, including a fully convolutional network network (FCN) to suggest the mitotic cell location and a fine discrimination model to refine the predictions. Li et al. [17] proposed a DeepMitosis framework with three stages. Firstly, bounding box labels were generated using FCN during training. Secondly, mitotic cells were localized using Faster R-CNN. Thirdly, the detection patches were then classified using ResNet to accurately detect mitotic cells and remove false positives. Das et al. [10] proposed a CNN onto wavelet decomposed patches for mitotic cell detection and evaluated the method using MITOS (ICPR2012) and MITOS-ATYPIA-14 datasets. Cai et al. [5] constructed a modified regional CNN (R-CNN) to automatically detect mitotic figures. Alom et al. [1] developed MitosisNet for mitotic cell detection from histopathological images. Sohail et al. [21] built a four-stage MP-MitDet framework for mitotic cell detection in H&E hispathological slides, consisting of a label-refiner, tissue-level mitotic region selection, blob analysis and cell-level refinement.

However, the above-mentioned studies were limited to single type mitotic cell detection, and multi-type mitotic figure detection and classification is poorly explored. In 2019, Bertram et al. [3] utilized a deep neural network to detect and classify mitotic figures that were later assessed by two human experts to generate a large-scale dataset for mitotic figure assessment. Hence, there is a unmet need for automatic systems in multi-type mitotic figure detection and classification. As multi-type mitotic figure classification on WSI is poorly explored, five recently published approaches in mitotic figure detection [17, 5, 1, 21, 3] and the three best performing models of the recent MIDOG 2021 challenge [2] are adopted as benchmark methods for single type mitotic cell detection.

S2: Materials

S2.1: Bone marrow WSI dataset (with 19 cell types)

The bone marrow experimental data were built from the Department of Laboratory Medicine, National Taiwan University Hospital, and an ethical approval has been obtained from the research ethics committee of the National Taiwan University Hospital (201911074RINB). Each slide was stained with Liu's stain, and WSIs were generated with 400x overall magnification using 3DHISTECH whole-slide scanner (Pannoramic 250 Flash III). The averaged digital size and physical size of individual slides are 253,070×482,247 pixels and 21.83×41.59 mm^2, respectively. The reference standard, containing 16,456 annotations, was produced by two specialized medical experts, involving one medical doctor with six years' experience and one medical examiner with more than 20 years' experience in BM examination. Figure S1 presents the distribution of the BM data set for each cell type built in this study.

S2.2: H&E Mitotic figure assessment WSI dataset (with five cell types)

Canine cutaneous mast cell tumor (CCMCT) dataset [3] was utilized, containing a total of 262,481 annotations, and 44,880 of which are mitotic figures. This dataset contains the largest number of annotated mitotic figures currently available. Figure S1 lists out the distribution of the whole set for each mitotic cell type used in this study. In evaluation, we utilize the same train-test split as presented in [3]. The CCMCT dataset consists of 32 WSIs; out of which 21 WSIs (66%) were used for training and 11 WSIs (34%) were used for testing the CW-Net. This data set could be downloaded from `https://github.com/DeepPathology/MITOS_WSI_CCMCT`.

S3: Discussion on System Limitation

Figure S2 presents the confusion matrix of inter-observer analysis between the two examiners, showing that even medical experts render much higher consistent decisions on Mitotic than Blast and that Blast cells tend to be confused with Proerythroblast cells. Figure S3 displays some sample cell images of Blast, AI misclassified cases, Proerythroblast and Mitotic cells, showing that the appearances of Blast and Proerythroblast cells are quite similar. In comparison, Mitotic cells present distinctive image patterns, and the two examiners produce perfectly matched decisions. This may be the reason why the proposed system obtains lower recall in Blast than in Mitotic cells.

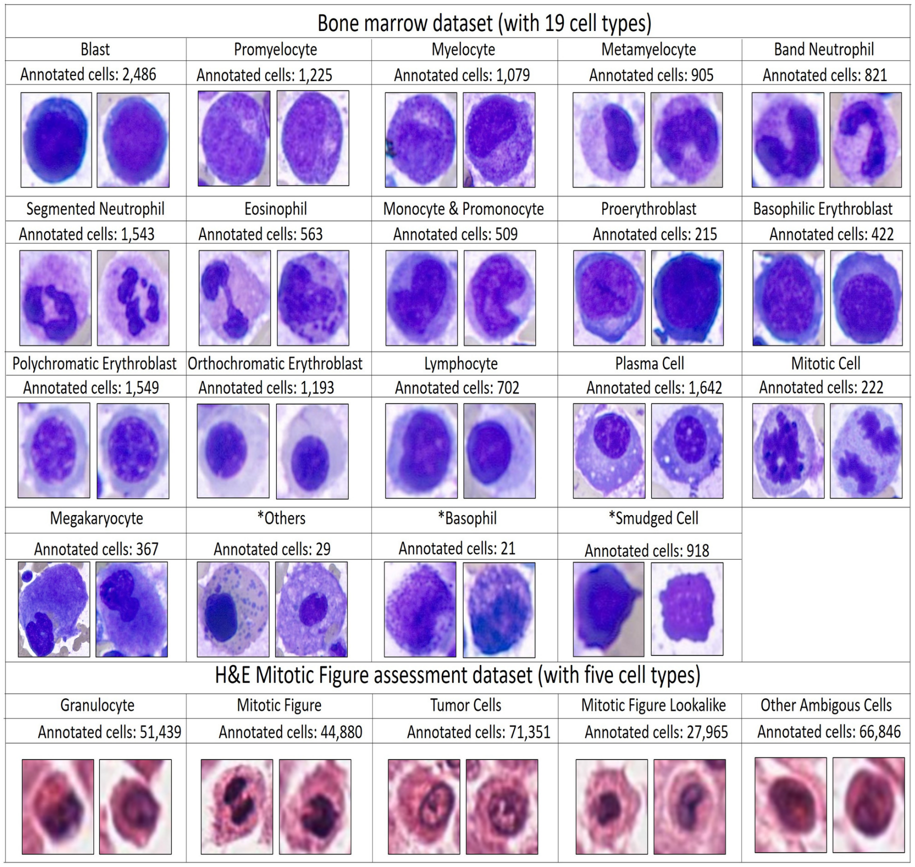

* These cell types are not included in the training set. Others include histiocyte/macrophage, osteoblast, osteoclast and non-hematopoietic cell.

Figure S1: Sample cells with the number of the annotated cells of various cell types in the two datasets used in this study.

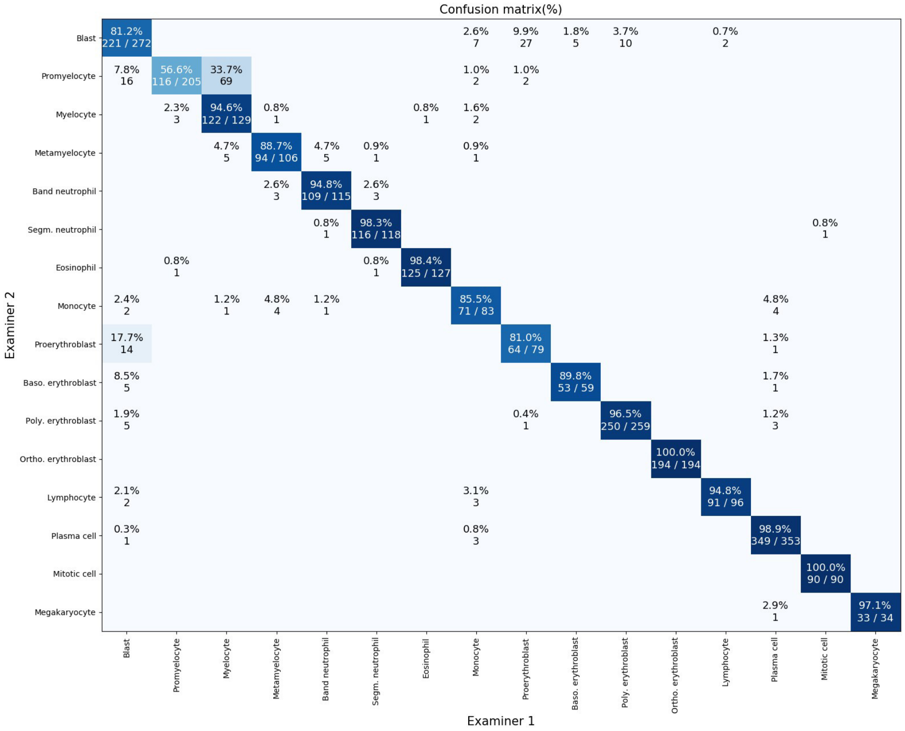

Figure S2: The detailed confusion matrix of the inter-observer analysis between the two examiners.

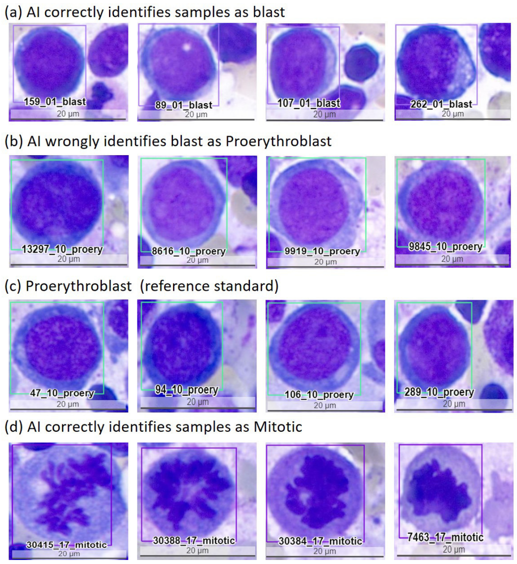

Figure S3: Sample images of BLAST, misclassified cases, Proerythroblast and Mitotic cells.

S4: Intra- and Inter-observer Reliability Analysis

For the intra-observer variability, we performed the kappa analysis on 665 randomly selected BM cells from three WSIs using two sets of annotations produced by each medical expert at an interval of one week, and the second annotation set was not used for further inter-observer variability analysis. For the inter-observer variability, we performed the kappa analysis on a larger set of ($\approx$ 2000) randomly selected BM cells from five WSIs. Supplementary Table S1 presents the Cohen's kappa statistical analysis results. In addition, the confusion matrices with detailed numbers and percentages are presented in the Supplementary Figure S4.

For the inter-observer analysis between AI and examiners, high kappa values of 0.824 and 0.908 were obtained, showing that the proposed AI model is reliable and highly consistent to the specialized medical examiners' decisions. Moreover, the results show that the second examiner who has more than 20 years of expertise in BM NDC analysis produces more consistent decisions, obtaining higher intra-observer kappa than the first examiner. In addition, the results of inter-observer analysis show that the mean kappa between the proposed AI model and the senior examiner is higher than the one with the junior examiner.

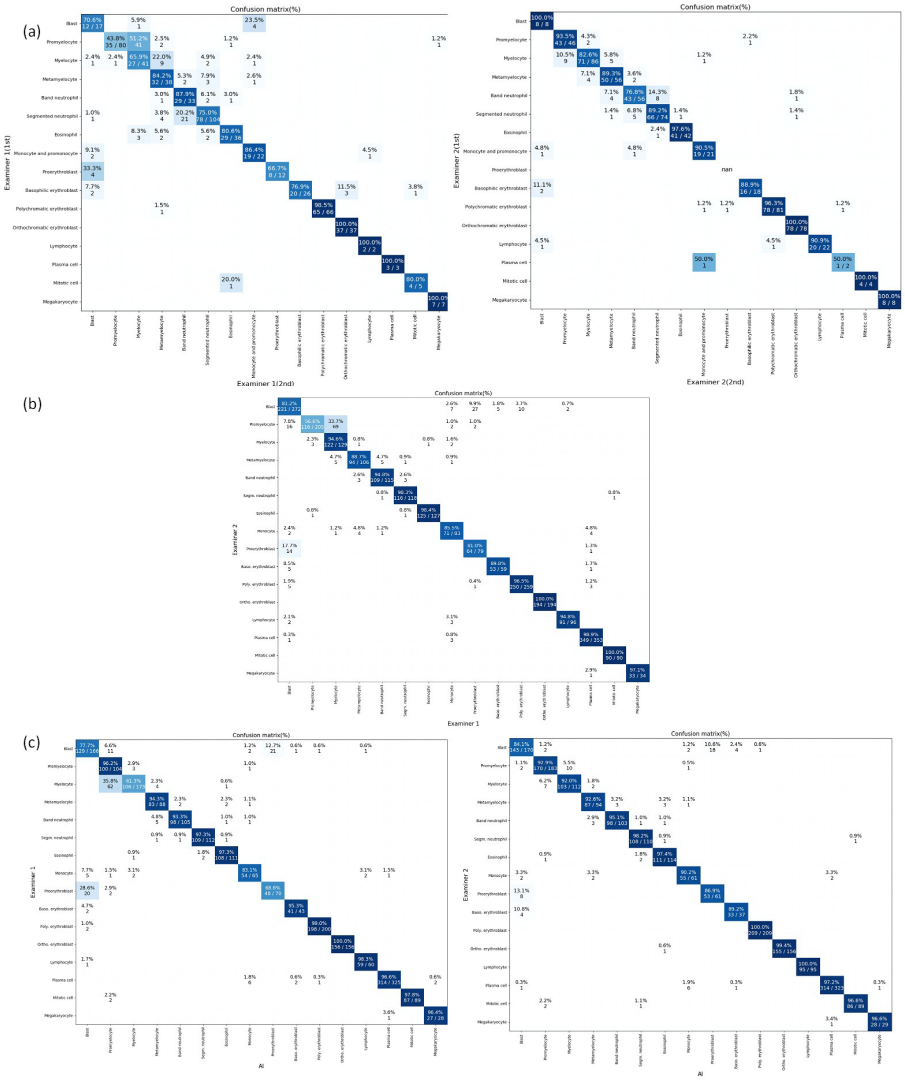

Figure S4: Confusion matrices for (a) intra-observer analysis, (b) inter-observer analysis between the two examiners and (c) inter-observer analysis between the AI prediction by the proposed CW-Net and individual examiners.

Table S1: Intra- and inter-observer reliability analysis using Cohen's kappa

Intra-observer analysis on 665 cells	
	Mean kappa
Intra-examiner 1	0.608 (good agreement)
Intra-examiner 2	0.789 (good agreement)
Inter-observer analysis on $\approx$ 2000 cells	
	Mean kappa
Inter-examiner 1 vs. 2	0.8 (good agreement)
Inter-AI vs. examiner 1	0.824 (excellent agreement)
Inter-AI vs. examiner 2	0.908 (excellent agreement)

References

[1] Alom, M. Z., T. Aspiras, T. M. Taha, T. Bowen, and V. K. Asari (2020). Mitosisnet: end-to-end mitotic cell detection by multi-task learning. *IEEE Access 8*, 68695–68710.

[2] Aubreville, M., N. Stathonikos, C. A. Bertram, R. Klopfleisch, N. Ter Hoeve, F. Ciompi, F. Wilm, C. Marzahl, T. A. Donovan, A. Maier, et al. (2023). Mitosis domain generalization in histopathology images—the midog challenge. *Medical Image Analysis 84*, 102699.

[3] Bertram, C. A., M. Aubreville, C. Marzahl, A. Maier, and R. Klopfleisch (2019). A large-scale dataset for mitotic figure assessment on whole slide images of canine cutaneous mast cell tumor. *Scientific data 6*(1), 1–9.

[4] Bilen, H. and A. Vedaldi (2016). Weakly supervised deep detection networks. In *2016 IEEE Conference on Computer Vision and Pattern Recognition (CVPR)*, pp. 2846–2854.

[5] Cai, D., X. Sun, N. Zhou, X. Han, and J. Yao (2019). Efficient mitosis detection in breast cancer histology images by rcnn. In *2019 IEEE 16th International Symposium on Biomedical Imaging (ISBI 2019)*, pp. 919–922. IEEE.

[6] Cao, C., Y. Huang, Y. Yang, L. Wang, Z. Wang, and T. Tan (2019). Feedback convolutional neural network for visual localization and segmentation. *IEEE Transactions on Pattern Analysis and Machine Intelligence 41*(7), 1627–1640.

[7] Chandradevan, R., A. Aljudi, B. Drumheller, N. Kunananthaseelan, M. Amgad, D. Gutman, L. Cooper, and D. Jaye (2019, 09). Machine-based detection and classification for bone marrow aspirate differential counts: initial development focusing on nonneoplastic cells. *Laboratory Investigation 100.*

[8] Chen, H., Q. Dou, X. Wang, J. Qin, and P. A. Heng (2016). Mitosis detection in breast cancer histology images via deep cascaded networks. In *Thirtieth AAAI conference on artificial intelligence.*

[9] Choi, J. W., Y. Ku, B. W. Yoo, J.-A. Kim, D. S. Lee, Y. J. Chai, H.-J. Kong, and H. C. Kim (2017). White blood cell differential count of maturation stages in bone marrow smear using dual-stage convolutional neural networks. *PloS one 12*(12).

[10] Das, D. K. and P. K. Dutta (2019). Efficient automated detection of mitotic cells from breast histological images using deep convolution neutral network with wavelet decomposed patches. *Computers in biology and medicine 104*, 29–42.

[11] Ghosh, P., D. Bhattacharjee, and M. Nasipuri (2016). Blood smear analyzer for white blood cell counting: a hybrid microscopic image analyzing technique. *Applied Soft Computing 46*, 629–638.

[12] Goodfellow, I., J. Pouget-Abadie, M. Mirza, B. Xu, D. Warde-Farley, S. Ozair, A. Courville, and Y. Bengio (2014). Generative adversarial nets. In Z. Ghahramani, M. Welling, C. Cortes, N. D. Lawrence, and K. Q. Weinberger (Eds.), *Advances in Neural Information Processing Systems 27*, pp. 2672–2680. Curran Associates, Inc.

[13] Hu, B., Y. Tang, E. I. Chang, Y. Fan, M. Lai, and Y. Xu (2019, May). Unsupervised learning for cell-level visual representation in histopathology images with generative adversarial networks. *IEEE Journal of Biomedical and Health Informatics 23*(3), 1316–1328.

[14] Hu, Y., M. Modat, E. Gibson, W. Li, N. Ghavami, E. Bonmati, G. Wang, S. Bandula, C. M. Moore, M. Emberton, et al. (2018). Weakly-supervised convolutional neural networks for multimodal image registration. *Medical image analysis 49*, 1–13.

[15] Jiao, L., F. Zhang, F. Liu, S. Yang, L. Li, Z. Feng, and R. Qu (2019). A survey of deep learning-based object detection. *IEEE Access 7*, 128837–128868.

[16] Kimura, K., Y. Tabe, T. Ai, I. Takehara, H. Fukuda, H. Takahashi, T. Naito, N. Komatsu, K. Uchihashi, and A. Ohsaka (2019). A novel automated image analysis system using deep convolutional neural networks can assist to differentiate mds and aa. *Scientific reports 9*(1), 1–9.

[17] Li, C., X. Wang, W. Liu, and L. J. Latecki (2018). Deepmitosis: Mitosis detection via deep detection, verification and segmentation networks. *Medical image analysis 45*, 121–133.

[18] Mishra, S., B. Majhi, P. K. Sa, and L. Sharma (2017). Gray level co-occurrence matrix and random forest based acute lymphoblastic leukemia detection. *Biomedical Signal Processing and Control 33*, 272–280.

[19] Reta, C., L. Altamirano, J. A. Gonzalez, R. Diaz-Hernandez, H. Peregrina, I. Olmos, J. E. Alonso, and R. Lobato (2015). Segmentation and classification of bone marrow cells images using contextual information for medical diagnosis of acute leukemias. *PloS one 10*(6).

[20] Sahlol, A. T., P. Kollmannsberger, and A. A. Ewees (2020). Efficient classification of white blood cell leukemia with improved swarm optimization of deep features. *Scientific Reports 10*(1), 1–11.

[21] Sohail, A., A. Khan, N. Wahab, A. Zameer, and S. Khan (2021). A multi-phase deep cnn based mitosis detection framework for breast cancer histopathological images. *Scientific Reports 11*(1), 1–18.

[22] Wang, C.-W., S.-C. Huang, Y.-C. Lee, Y.-J. Shen, S.-I. Meng, and J. L. Gaol (2022). Deep learning for bone marrow cell detection and classification on whole-slide images. *Medical Image Analysis 75*, 102270.

[23] Wang, X., X. Deng, Q. Fu, Q. Zhou, J. Feng, H. Ma, W. Liu, and C. Zheng (2020). A weakly-supervised framework for covid-19 classification and lesion localization from chest ct. *IEEE Transactions on Medical Imaging*.

[24] Xie, Y., F. Xing, X. Shi, X. Kong, H. Su, and L. Yang (2018). Efficient and robust cell detection: A structured regression approach. *Medical Image Analysis 44*, 245 – 254.

[25] Yang, Z., D. Mahajan, D. Ghadiyaram, R. Nevatia, and V. Ramanathan (2019). Activity driven weakly supervised object detection. In *Proceedings of the IEEE Conference on Computer Vision and Pattern Recognition*, pp. 2917–2926.

[26] Yu, T.-C., W.-C. Chou, C.-Y. Yeh, C.-K. Yang, S.-C. Huang, F. M. Tien, C.-Y. Yao, C.-L. Cheng, M.-K. Chuang, H.-F. Tien, Q.-Y. Zhang, W.-H. Hsu, and S.-C. Chou (2019, 11). Automatic bone marrow cell identification and classification by deep neural network. *Blood 134*, 2084–2084.
